# Early supplementation with zinc proteinate does not change rectal microbiota but increases growth performance by improving antioxidant capacity and plasma zinc concentration in preweaned dairy calves

**DOI:** 10.3389/fvets.2023.1236635

**Published:** 2023-09-27

**Authors:** Junhao Liu, Xin Yu, Fengtao Ma, Yeqianli Wo, Yuhang Jin, Nesrein M. Hashem, Peng Sun

**Affiliations:** ^1^State Key Laboratory of Animal Nutrition and Feeding, Institute of Animal Science, Chinese Academy of Agricultural Sciences, Beijing, China; ^2^Department of Animal and Fish Production, Faculty of Agriculture, Alexandria University, Alexandria, Egypt

**Keywords:** zinc proteinate, zinc oxide, growth performance, antioxidant capacity, plasma zinc concentration

## Abstract

The present study evaluated the effects of early supplementation with zinc proteinate (ZnP) or zinc oxide (ZnO) for 2 weeks on the growth performance, redox status, plasma trace element concentrations, and rectal microbiota of preweaned dairy calves. A total of 60 newborn healthy female Holstein dairy calves, with initial body weight (BW): 41.33 ± 0.62 kg, were randomly allocated to 5 groups of 12 each: a control group (CON); three groups supplemented with 261 (L-ZnP), 523 (M-ZnP), and 784 (H-ZnP) mg/day ZnP, equivalent to 40, 80, and 120 mg/day zinc, respectively; and one group supplemented with 232 mg/day ZnO, equivalent to 180 mg/day zinc (ZnO). Zinc supplements were administered on days 1–14, and the calves were followed up until day 70. Zinc supplementation increased total dry matter intake (DMI) and starter DMI compared with the CON group (*p* < 0.01). The final BW, average daily gain, and feed efficiency were higher in the M-ZnP, H-ZnP, and ZnO groups (*p* < 0.05). The incidence of diarrhea on days 1–28 was reduced by zinc administration (*p* < 0.01), whereas the incidence on days 29–56 was lower in the M-ZnP and ZnO groups (*p* < 0.05). Serum glutathione peroxidase activity, total antioxidant capacity, immunoglobulin G and plasma zinc concentrations were increased linearly (*p* < 0.05), while the serum concentration of malondialdehyde was decreased linearly (*p* < 0.01), as the dose of ZnP increased. ZnP yielding 80 mg/day zinc had similar effects as ZnO yielding 180 mg/day zinc, except that final BW was higher in the ZnO group (*p* < 0.05). At the phylum level, ZnO decreased the relative abundance of Firmicutes while increasing the abundance of Bacteroidetes (*p* < 0.05). At the genus level, ZnO increased the relative abundances of *Prevotella*, *Subdoligranulum*, and *Odoribacter* (*p* < 0.05). These findings indicated that early supplementation with ZnP did not affect the rectal microbiota of preweaned dairy calves but increased their growth performance, antioxidant capacity, and plasma zinc concentration. In summary, ZnP is an organic zinc source with greater bioavailability than ZnO for preweaned dairy calves. Early dietary supplementation with ZnP yielding 80 mg/day zinc is recommended.

## 1. Introduction

The health of calves is of great importance in maintaining optimal lactation performance of adult dairy cows and the sustainable development of pastures. In traditional breeding, the lactation period of calves is generally 3–6 months; weaning earlier than 6 weeks has been found to reduce average daily gain (ADG) and increase morbidity, thus reducing the lifespan of dairy cows (1). To shorten the lactation cycle, most farms have begun to implement early weaning. However, the high levels of stress caused by early weaning may have lasting effects on calf health, which may reduce future production parameters, including growth, lactation, and reproductive performance, and in severe cases may lead to calf death (2, 3).

Efforts are therefore required to minimize the adverse effects of weaning, such as reducing stress associated with weaning or disease risk, and improving feed conversion efficiency. Antibiotics once played important roles in these areas (4, 5). However, the intensive use of synthetic antibiotics raises global concerns due to the increased health risks related to antimicrobial resistance (6). Therefore, it is imperative to seek appropriate feed additives to reduce the possible damage caused by weaning.

Zinc is an essential nutrient element and is involved in many physiologic functions in the body (7). Dietary zinc supplementation has been found to increase growth performance, promote antioxidant and immune activities, improve the intestinal microflora, and reduce the incidence of diarrhea (8–10). Although oral administration of large amounts of zinc oxidant (ZnO) was often used to promote growth and reduce diarrhea in young animals (11), unabsorbed zinc secreted in feces may post a threat to the environment. Thus, in 2017, the Ministry of Agriculture and Rural Affairs of China banned the supplementation of animal diets with high levels of ZnO and mandated that the level of zinc in calf diets not exceed 180 mg/kg.

Organic zinc has a higher bioavailability than inorganic zinc (12, 13). There are many commercial sources of organic zinc such as zinc methionine, zinc polysaccharide, zinc lysine, zinc amino acid chelate, and zinc proteinate, etc. Ma et al. (14) suggested that zinc methionine supplementation lowers the incidence of diarrhea in postnatal Holstein dairy calves by reducing intestinal permeability, which confirms the effectiveness of organic zinc as a potential treatment for diarrhea during early life calf rearing. Comparable to the same dose of zinc from zinc methionine, our previous study showed that supplementation with ZnP to yield 80 mg/day zinc also reduced the incidence of diarrhea and increased the growth performance and immune function of dairy calves (15). It is known that ZnP is a novel form of organic zinc prepared from soybean protein isolate and feed-grade inorganic zinc (16). The ZnP supplementation was found to significantly enhance humoral immunity, and superoxide dismutase (SOD) activity in red blood cells (17). Therefore, we hypothesized that early supplementation with ZnP during the first 2 weeks of life may alleviate diarrhea and improve the growth performance of preweaned dairy calves by improving their antioxidant capacity, immune function, and rectal microbial microbiota. The present study was designed to determine the optimal dose of ZnP for dairy calves and compare that dose of ZnP with ZnO yielding 180 mg/day zinc. Outcomes analyzed included growth performance, the incidence of diarrhea, serum concentrations of antioxidants and immune indicators, plasma concentrations of trace elements, and rectal microbiota of preweaned Holstein dairy calves, which might achieve the most optimal dose of ZnP that can maintain calf health and minimize the release of zinc into the environment.

## 2. Materials and methods

This study was performed at the Yanqing Breeding Farm of the Beijing Dairy Cattle Center (Beijing, China). The calves were maintained according to the standards of the Chinese Academy of Agricultural Sciences Animal Care and Use Committee.

### 2.1. Animals and diet

The study included 60 newborn healthy female Holstein dairy calves of initial mean body weight (BW) 41.33 ± 0.62 kg. Each calf was fed 4 L of colostrum from a bottle within 1 h of birth, followed by 1.5 L of raw milk three times per day from a bottle at 0600, 1400, and 1800 on days 2–4, and 4 L of raw milk twice per day from a bottle at 0600 and 1800 on days 5–70. Based on their growth condition, all calves were removed from their dams at age 4–7 days, housed in individual pens, and then fed with granulated starter produced by the Feed Branch of Beijing Shounong Animal Science and Technology Development Co., Ltd. (Beijing, China). The crude protein (CP; AOAC International, 2000; method 976.05), ether extract (EE; AOAC International, 2003; method 4.5.05), and dry matter content (DM; AOAC International, 2005; method 930.15) of starter was measured using the standard procedures of AOAC International (18–20). The neutral detergent fiber (NDF) and acid detergent fiber (ADF) contents were determined according to Van Soest et al. (21). The density, milk protein, milk fat, lactose, total solids (TS), and solids non-fat (SNF) were determined by a milk composition analyzer (MilkoScanTM FT6000, FOSS Corporation, Denmark). The nutrient compositions of the milk and starter are presented in Table 1. The concentrations of zinc in water, milk, and starter were 0, 3.35 mg/kg and 175 mg/kg DM, respectively.

**Table 1:** Nutrient composition of the milk and starter.^a^

Item	Milk	Starter (DM basis)
CP, %	—	24.45
Ether extract, %	—	1.68
Ash, %	—	7.75
NDF, %	—	12.20
ADF, %	—	6.85
DM, %	—	89.04
Zinc, mg/kg	3.35	175.00
Milk protein, %	3.25	—
Milk fat, %	4.02	—
Total solids, %	12.56	—
Lactose, %	4.91	—
Density, g/mL	1.03	—

CP, crude protein; NDF, neutral detergent fiber; ADF, acid detergent fiber; DM, dry matter.

a Analyzed value.

### 2.2. Experimental design and sample collection

The calves were randomly allocated to 5 groups of 12 using a random number generator (Microsoft Corp., WA, *n* = 12 each): a negative control group without extra zinc supplementation (CON group); three groups supplemented with 261 (L-ZnP group), 523 (M-ZnP group), and 784 (H-ZnP group) mg/day ZnP, equivalent to 40, 80, and 120 mg/day zinc, respectively; and one group supplemented with 232 mg/d ZnO, equivalent to 180 mg/day zinc (ZnO group). These doses were based on previous studies (15, 22, 23). The ZnP (*Q*_f_ = 327) was donated by the Mineral Nutrition Research Division, Institute of Animal Science, Chinese Academy of Agricultural Sciences (Beijing, China). The ZnO or ZnP was administered from birth until 14 days of age. The appropriate amounts of ZnO or ZnP were mixed with 200 mL of raw milk and fed to the calves, followed by feeding with additional raw milk. The calves were monitored from birth to 70 days of age, during which time they had free access to water and starter.

Blood samples were collected from the external jugular vein of each calf using vacutainer tubes, with or without heparin sodium (BD Biosciences, San Jose, CA), before morning feedings on days 15, 29, 43, 57, and 71 after birth. Serum and plasma were prepared by centrifugation at 3000 × g for 15 min at 4°C and then stored at −20°C for subsequent analyses.

Fecal samples were randomly collected from the rectum of 8 calves in each group on the morning of day 71. About 0.2 g of fecal sample from each animal was collected in a 2 mL tube and stored at −80°C for subsequent analysis of the microbiota.

### 2.3. Growth performance and incidence of diarrhea

The BW of each calf was recorded before morning feedings on days 1, 15, 29, 43, 57, and 71 after birth. ADGs were calculated for these time intervals and for the entire length of the study. Daily intake of milk and starter was recorded, and the total dry matter intake (DMI), starter DMI, and feed to gain ratio (F:G) were calculated for each calf.

During the trial period, the feces of each calf were monitored twice daily, in the morning and evening, and scored. Normal and cylindrical feces were scored as 1 point; slight, thin, soft, and tangible feces as 2 points; shapeless stools with high moisture content as 3 points; and liquid, shapeless, watery feces as 4 points (24). Scores of 3 and 4 for 2 consecutive days were defined as diarrhea, and the incidence of diarrhea was recorded as described (25).

### 2.4. Concentrations of serum immunoglobulins, antioxidant indicators and plasma trace elements

The concentrations of serum immunoglobulin (Ig) A, IgG, and IgM were measured using bovine ELISA kits (Wuhan ColorfulGene Biological Technology; Wuhan, China) according to the manufacturer’s instructions.

Glutathione peroxidase (GSH-Px) and SOD activities, and total antioxidant capacity (T-AOC) and malondialdehyde (MDA) concentrations, were analyzed by radioimmunoassays using commercially available kits (Nanjing Jian Cheng Bioengineering Institute, Nanjing, China) in accordance with the manufacturer’s instructions.

The concentrations of copper (Cu), iron (Fe), zinc (Zn), calcium (Ca), and magnesium (Mg) in plasma were determined by inductively coupled plasma optical emission spectroscopy (ICP-OES), according to the Chinese National Standards (GB 5009.268, China, 2016) with some modifications (26).

### 2.5. Extraction of total DNA from rectal microbes and *16S rRNA* gene sequencing

The DNA was extracted from rectal contents using M5635-02 Mag-Bind Soil DNA kits (Omega Biotek, United States), according to the manufacturer’s instructions. The quality of the extracted DNA was determined by 0.8% agarose gel electrophoresis, and the quantity of the DNA were assessed using a NanoDrop NC2000 spectrophotometer (Thermo Fisher Scientific, Waltham, MA).

DNA sequences were PCR amplified using Applied Biosystems^™^ 2,720 Thermal Cycler (Thermo Fisher Scientific, Waltham, MA), Pfu high-fidelity DNA polymerase (Beijing Quanshijin Biotechnology Co., Ltd., China), and the primers 338F (5′-ACTCCTACGGGAGGCAGCA-3′) and 806R (5′-GGACTACHVGGGTWTCTAAT-3′) (Personalbio Biotechnology Co., Ltd.; Shanghai, China). Each 25 μL PCR reaction mixture contained 5 μL of 5 × reaction buffer, 5 μL of 5 × GC buffer, 2 μL of dNTPs (2.5 mM each), 1 μL each of the forward and reverse primers (10 μM each), 2 μL of template DNA, 8.75 μL of ddH_2_O, and 0.25 μL of Q5 DNA polymerase. The sequencing area was “16s-V3-V4,” and all samples were assayed in triplicate.

The amplification protocol consisted of an initial denaturation at 98°C for 2 min, followed by 25–30 cycles of denaturation at 98°C for 15 s, annealing at 55°C for 30 s, and extension at 72°C for 5 min, followed by a final extension at 72°C for 5 min. The amplification products were electrophoresed on 2% agarose gels, and the target fragments were extracted from the gels using a DNA purification kit (Axygen, Biosciences, Union City, CA). Depending on the results of electrophoresis, the PCR products were amplified using Quant-iT PicoGreen dsDNA Assay Kits and quantified using a microplate reader (BioTek, FLx80). According to the fluorescent quantitative results, the samples were mixed in equal quantities and used to construct a sequencing library using an Ilumina Truseq Nano DNA LT Library Prep Kit. Finally, the V3–V4 amplicons were sequenced using the paired-end method on an Illumina Miseq at Majorbio (Shanghai personalbio, Shanghai, China). The sequences have been registered at GenBank under accession number SRP320638.

### 2.6. Sequence analysis

The DADA2 method was used for primer removal, mass filtering, denoising, splicing, and chimera removal (27), followed by detailed processing (25). Richness was estimated using the Chao1 and observed species indices, and community diversity was assessed using the Shannon and Simpson indices. Beta diversity was analyzed using Bray–Curtis distance and visualized by principal coordinates analysis (PCoA).

### 2.7. Statistical analysis

The incidence of diarrhea was compared among groups using the chi-squared contingency test. Growth performance and blood indices were compared using the MIXED procedure in SAS software (version 9.4, SAS Institute Inc., Cary, NC). A repeated measures model evaluated the random effect of calf and the fixed effects of treatment, day, and their interaction was used. Contrasts were performed to evaluate treatment effects, ZnP supplementation levels, and zinc source (M-ZnP vs. ZnO). The data are expressed as the least squares means and the standard error of the mean (SEM). Differences between treatment groups were evaluated using Tukey’s multiple range tests. Differences in rectal microbial alpha diversity and the relative abundances of species at different levels were analyzed by Kruskal-Wallis tests using SPSS 20 software. The range of *R*- and *p*-values selected by the correlation heat map was >0.3 and <0.05, respectively. Correlations of the populations of the principal fecal microbes with growth performance, antioxidant indicators, and plasma zinc concentrations on day 70 were analyzed by Spearman’s correlation method on Genescloud.^1^ *p*-values <0.05 were defined as statistically significant, whereas *p*-values of 0.05–0.10 were considered significant tendencies.

## 3. Results

### 3.1. Growth performance and incidence of diarrhea

The effects of different zinc sources on growth performance and incidence of diarrhea in preweaned Holstein dairy calves are presented in Table 2. At baseline, BW was similar in the five groups of calves. However, supplementation with ZnP or ZnO increased starter DMI and total DMI compared with the CON group (*p* < 0.01). As the level of ZnP supplementation increased, starter DMI and total DMI increased linearly and quadratically, and peaked in the M-ZnP group (*p* < 0.05). The final BW, ADG, and feed efficiency also increased linearly (*p* < 0.05), being significantly higher in the M-ZnP, H-ZnP, and ZnO groups than in the CON group (*p* < 0.05). Growth performance was not affected by zinc source except for the final BW, which was significantly higher in the ZnO than in the M-ZnP group (*p* < 0.05). Supplementation with ZnP or ZnO reduced the incidence of diarrhea on days 1–28 (*p* < 0.01), whereas, on days 29–56, the incidence of diarrhea was lower in the M-ZnP and ZnO groups than in the CON group (*p* < 0.05). The growth performance of the calves varied significantly with age, with day and the treatment × day interaction having significant effects on total DMI and feed to gain ratio (*p* < 0.05).

**Table 2:** Effects of different zinc sources and concentrations on growth performance and incidence of diarrhea of preweaned Holstein dairy calves.

Item^a^	Treatment^b^					SEM	*p*-value					
	CON	L-ZnP	M-ZnP	H-ZnP	ZnO		Linear	Quadratic	Source	Trt	Day	Trt × day
Initial BW (kg)	40.08	43.08	42.11	41.42	40.00	1.38	—	—	—	0.461	—	—
Final BW (kg)	99.19^c^	100.81^c^	102.35^b^	103.53^ab^	103.99^a^	0.53	<0.01	0.681	0.033	<0.01	—	—
Average daily gain (ADG; g/d)	826.19^b^	852.98^ab^	872.62^a^	888.69^a^	893.45^a^	10.26	0.036	0.024	0.157	<0.01	<0.01	0.983
Total dry matter intake (total DMI; g/d)	1320.78^b^	1369.88^a^	1373.78^a^	1370.35^a^	1371.39^a^	11.21	<0.01	0.021	0.875	<0.01	<0.01	0.029
Starter dry matter intake (starter DMI; g/d)	343.18^b^	383.54^a^	387.00^a^	386.42^a^	382.51^a^	9.25	<0.01	0.033	0.725	<0.01	<0.01	0.063
Feed to gain (F:G; g of DMI/g of gain)	1.71^a^	1.68^ab^	1.61^b^	1.63^ab^	1.60^b^	0.02	0.042	0.386	0.771	0.008	<0.01	0.004
Incidence of diarrhea (1–28 days; %)	18.2^a^	6.55^b^	3.57^b^	3.27^b^	4.17^b^	—	—	—	—	<0.01	—	—
Incidence of diarrhea (29–56 days; %)	4.17^a^	2.08^abc^	0.60^c^	2.98^ab^	1.49^bc^	—	—	—	—	0.048	—	—
Incidence of diarrhea (57–70 days; %)	4.17	3.57	1.79	1.79	2.98	—	—	—	—	0.616	—	—

^a,b,c^Significant differences within the same row (*p* < 0.05).

a BW, body weight; ADG, average daily gain; total DMI, total dry matter intake; starter DMI, starter dry matter intake; F:G, feed to gain.

b CON, control group (*n* = 12, no zinc proteinate or zinc oxide supplementation); L-ZnP, low-zinc proteinate group (*n* = 12, 261 mg/day of zinc proteinate); M-ZnP, medium-zinc proteinate group (*n* = 12, 523 mg/day of zinc proteinate); H-ZnP, high-zinc proteinate group (*n* = 12, 784 mg/day of zinc proteinate); ZnO, zinc oxide group, (*n* = 12, 232 mg/day of zinc oxide).

### 3.2. Concentrations of serum immunoglobulins, antioxidant indicators and plasma trace elements

The effects of different zinc sources on serum immune indices are shown in Table 3. Neither zinc supplementation nor zinc source affected the serum IgA, IgG, and IgM concentrations in these dairy calves. However, serum IgG concentration increased linearly with increasing ZnP dose (*p* < 0.05).

**Table 3:** Effects of different zinc sources and concentrations on serum immune indices of preweaned Holstein dairy calves.

Item^a^	Treatment^b^					SEM	*p*-value					
	CON	L-ZnP	M-ZnP	H-ZnP	ZnO		Linear	Quadratic	Source	Trt	Day	Trt × day
IgA, μg/mL	57.19	56.89	56.37	58.20	57.77	1.17	0.625	0.369	0.401	0.823	<0.01	0.972
IgG, mg/mL	13.60	13.75	14.01	14.26	14.23	0.24	0.035	0.864	0.529	0.223	<0.01	0.874
IgM, mg/mL	2.77	2.80	2.88	2.77	2.80	0.04	0.752	0.077	0.159	0.302	<0.01	0.428

a IgA, immunoglobulin A; IgG, immunoglobulin G; IgM, immunoglobulin M.

b CON, control group (*n* = 12, no zinc proteinate or zinc oxide supplementation); L-ZnP, low-zinc proteinate group (*n* = 12, 261 mg/day of zinc proteinate); M-ZnP, medium-zinc proteinate group (*n* = 12, 523 mg/day of zinc proteinate); H-ZnP, high-zinc proteinate group (*n* = 12, 784 mg/day of zinc proteinate); ZnO, zinc oxide group, (*n* = 12, 232 mg/day of zinc oxide).

Table 4 shows the effects of different zinc sources on serum antioxidant indices. The activities of serum GSH-Px (*p* < 0.01) and T-AOC (*p* < 0.05) increased linearly, whereas the serum concentrations of MDA (*p* < 0.01) decreased linearly, as the dose of ZnP increased. Furthermore, serum GSH-Px activity was higher in the ZnP and ZnO groups than in the CON group (*p* < 0.01), whereas the activity of SOD was higher in the M-ZnP and ZnO groups than in the CON and H-ZnP groups (*p* < 0.05). Zinc source did not significantly affect the serum antioxidant indices.

**Table 4:** Effects of different zinc sources and concentrations on antioxidant indices in the sera of preweaned Holstein dairy calves.

Item^a^	Treatment^b^					SEM	*p*-value					
	CON	L-ZnP	M-ZnP	H-ZnP	ZnO		Linear	Quadratic	Source	Trt	Day	Trt × day
MDA, nmol/mL	5.30	5.24	5.13	5.04	5.11	0.07	<0.01	0.872	0.849	0.068	<0.01	0.632
SOD, U/mL	80.23^c^	83.50^abc^	85.74^ab^	83.28^bc^	88.06^a^	1.65	0.134	0.088	0.332	0.018	<0.01	0.991
GSH-Px, U/mL	126.10^b^	129.82^a^	132.31^a^	132.72^a^	132.23^a^	1.20	<0.01	0.181	0.992	<0.01	<0.01	0.755
T-AOC, U/mL	3.73	3.86	4.08	4.05	4.07	0.10	0.012	0.44	0.958	0.052	<0.01	0.930

^a,b,c^Significant differences within the same row (*p* < 0.05).

a MDA, malondialdehyde; SOD, superoxide dismutase; GSH-Px, glutathione peroxidase; T-AOC, total antioxidant capacity.

b CON, control group (*n* = 12, no zinc proteinate or zinc oxide supplementation); L-ZnP, low-zinc proteinate group (*n* = 12, 261 mg/day of zinc proteinate); M-ZnP, medium-zinc proteinate group (*n* = 12, 523 mg/day of zinc proteinate); H-ZnP, high-zinc proteinate group (*n* = 12, 784 mg/day of zinc proteinate); ZnO, zinc oxide group, (*n* = 12, 232 mg/day of zinc oxide).

The effects of different zinc sources on plasma trace element concentrations are presented in Table 5. Neither zinc source nor zinc dose affected plasma Ca, Cu, Fe, and Mg concentrations. However, plasma zinc concentrations increased linearly as the dose of ZnP increased (*p* < 0.01). Plasma concentrations of zinc were significantly higher in the M-ZnP, H-ZnP, and ZnO groups than in the CON and L-ZnP groups (*p* < 0.01).

**Table 5:** Effects of different zinc sources and concentrations on plasma trace element concentrations of preweaned Holstein dairy calves.

Item	Treatment^a^					SEM	*p*-value					
	CON	L-ZnP	M-ZnP	H-ZnP	ZnO		Linear	Quadratic	Source	Trt	Day	Trt × day
Zinc, mg/L	1.33^c^	1.53^bc^	1.68^ab^	1.86^a^	1.89^a^	0.07	<0.01	0.852	0.051	<0.01	<0.01	<0.01
Calcium, mg/L	118.21	119.93	119.45	116.34	119.52	1.97	0.487	0.232	0.977	0.702	<0.01	0.113
Copper, mg/L	0.85	0.83	0.85	0.84	0.85	0.03	0.90	0.813	0.981	0.986	0.018	0.074
Iron, mg/L	1.76	1.64	1.88	1.42	2.25	0.27	0.533	0.531	0.339	0.285	<0.01	0.779
Magnesium, mg/L	21.38	22.13	21.83	21.93	23.34	0.66	0.65	0.630	0.118	0.323	<0.01	0.814

^a,b,c^Significant differences within the same row (*p* < 0.05).

a CON, control group (*n* = 12, no zinc proteinate or zinc oxide supplementation); L-ZnP, low-zinc proteinate group (*n* = 12, 261 mg/day of zinc proteinate); M-ZnP, medium-zinc proteinate group (*n* = 12, 523 mg/day of zinc proteinate); H-ZnP, high-zinc proteinate group (*n* = 12, 784 mg/day of zinc proteinate); ZnO, zinc oxide group, (*n* = 12, 232 mg/day of zinc oxide).

### 3.3. Rectal microbial diversity

**Figure 1:**
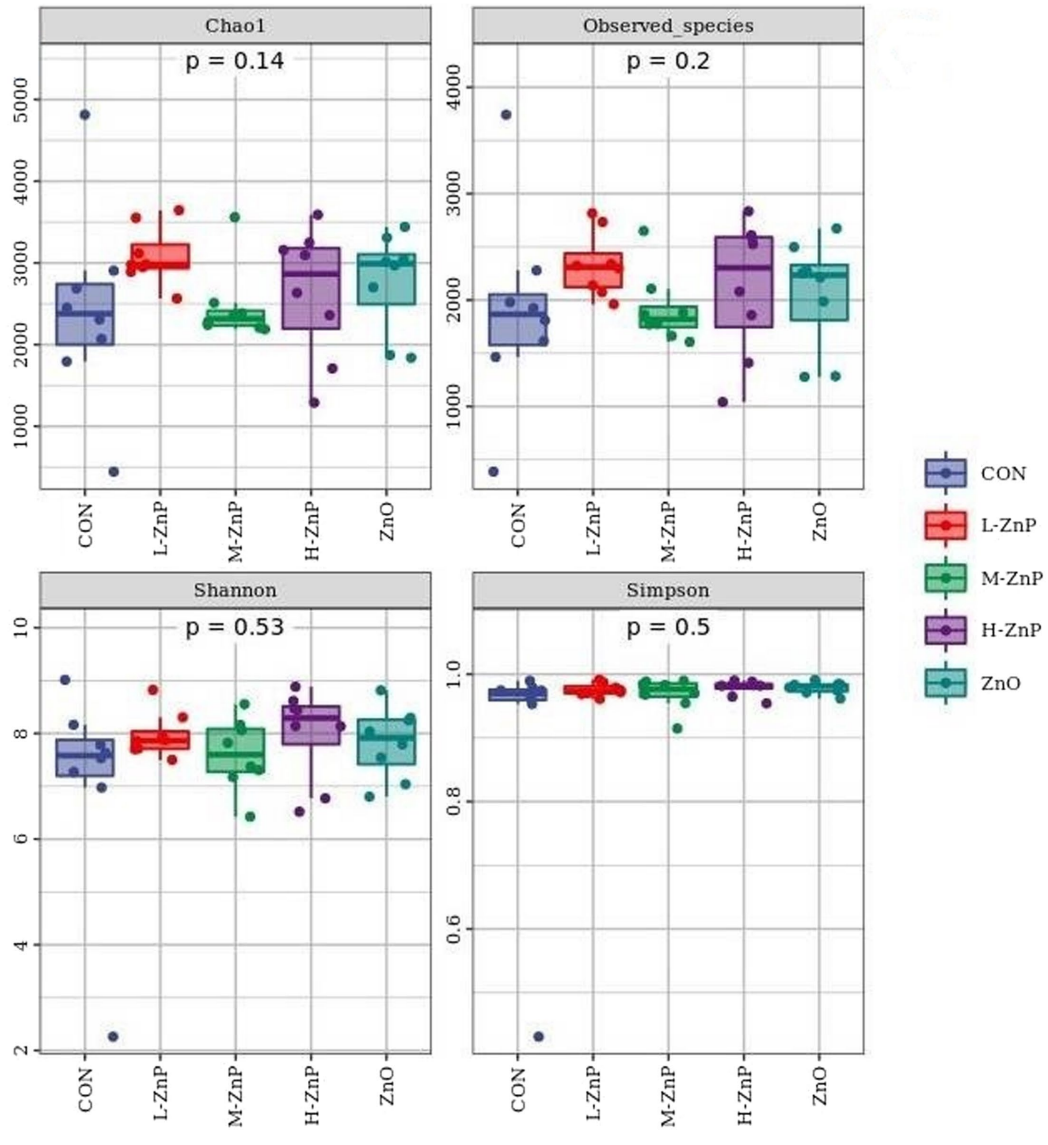
Effects of different zinc sources on rectal microbial α-diversity of Holstein dairy calves on day 70. CON, control group (*n* = 8, no zinc proteinate or zinc oxide supplementation); L-ZnP, low-zinc proteinate group (*n* = 8, 261 mg/day of zinc proteinate); M-ZnP, medium-zinc proteinate group (*n* = 8, 523 mg/day of zinc proteinate); H-ZnP, high-zinc proteinate group (*n* = 8, 784 mg/day of zinc proteinate); ZnO, zinc oxide group, (*n* = 8, 232 mg/day of zinc oxide).

**Figure 2:**
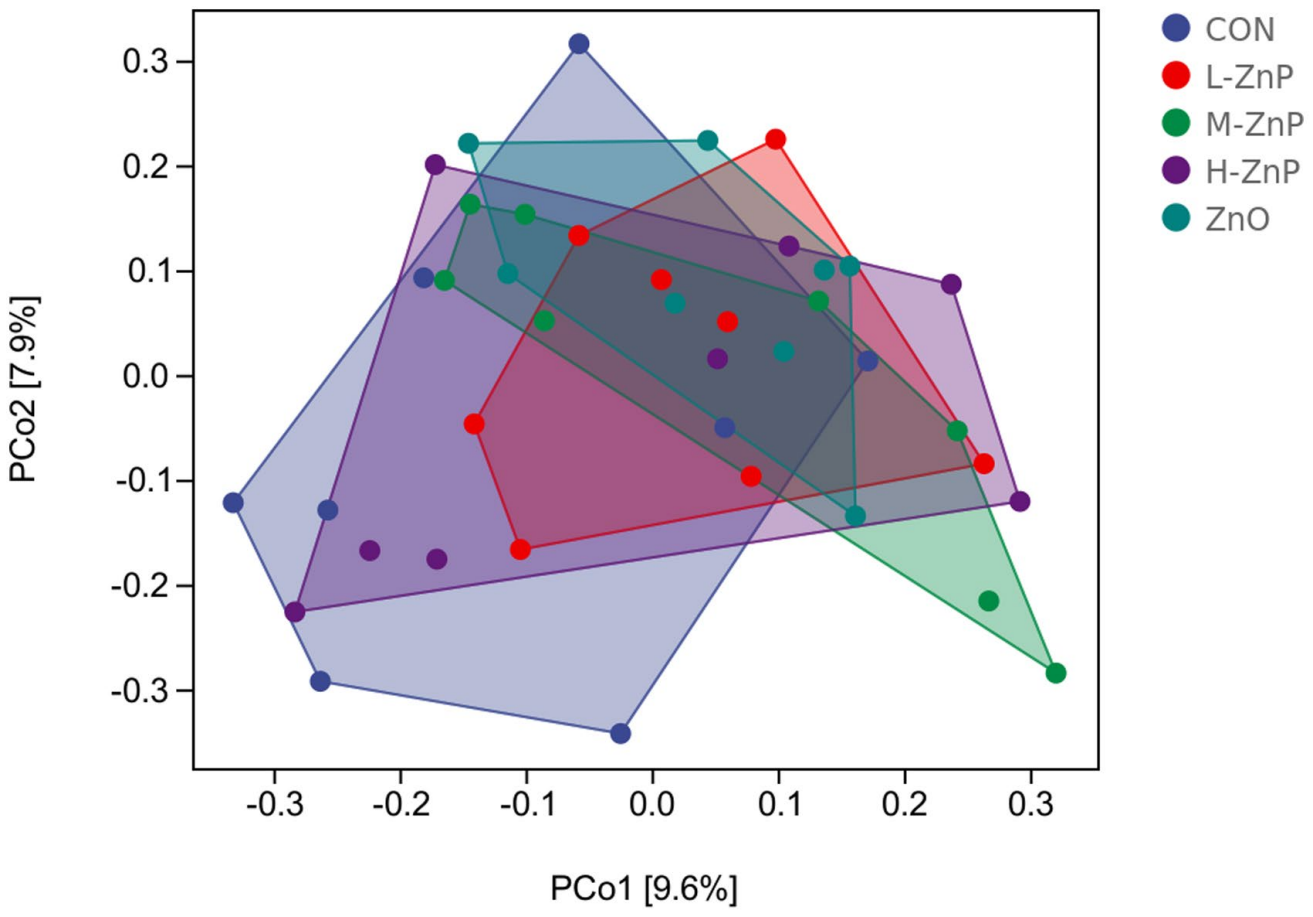
Principal coordinates analysis (PCoA) of the effects of different zinc sources on rectal microbiota of Holstein dairy calves on day 70. CON, control group (*n* = 8, no zinc proteinate or zinc oxide supplementation); L-ZnP, low-zinc proteinate group (*n* = 8, 261 mg/day of zinc proteinate); M-ZnP, medium-zinc proteinate group (*n* = 8, 523 mg/day of zinc proteinate); H-ZnP, high-zinc proteinate group (*n* = 8, 784 mg/day of zinc proteinate); ZnO, zinc oxide group, (*n* = 8, 232 mg/day of zinc oxide).

The effects of different zinc sources on rectal microbial diversity on day 70 are presented in Figures 1, 2. Indicators of α diversity showed that neither zinc supplementation nor zinc source affected the Chao1, Observed species, Shannon, and Simpson indices on day 70 (Figure 1). A plot of PCoA scores showed shifts of principal component between the CON and L-ZnP groups (*R* = 0.13, *p* = 0.048), the L-ZnP and ZnO groups (*R* = 0.24, *p* = 0.012), and the H-ZnP and ZnO groups (*R* = 0.21, *p* = 0.023) (Figure 2).

### 3.4. Rectal microbiota composition at the phylum level

The effects of different zinc sources on the rectal microbial composition at the phylum level on day 70 are shown in Table 6. Firmicutes and Bacteroidetes were the dominant phyla, followed by Actinobacteria, Proteobacteria, Cyanobacteria, and Fusobacteria. The relative abundance of Firmicutes was lower in the ZnO group than in the CON and H-ZnP groups (*p* < 0.05). In contrast, the relative abundance of Bacteroidetes was higher in the ZnO group than in the CON and L-ZnP groups (*p* < 0.05).

**Table 6:** Analysis of the principal rectal phyla in preweaned Holstein dairy calves on day 70.

Phylum, %	Treatment^a^					SEM	*p*-value
	CON	L-ZnP	M-ZnP	H-ZnP	ZnO		
Firmicutes	63.43^a^	45.57^ab^	52.46^ab^	62.14^a^	39.81^b^	0.18	0.048
Bacteroidetes	33.54^b^	33.69^b^	44.63^ab^	51.43^ab^	58.05^a^	0.19	0.042
Actinobacteria	1.38	0.85	1.76	2.60	0.84	0.02	0.987
Proteobacteria	0.93	1.15	0.61	0.99	0.87	0.01	0.342
Cyanobacteria	0.02	0.11	0.02	0.12	0.13	0.00	0.128
Fusobacteria	0.20	0.50	0.10	0.09	0.00	0.01	0.411

^a,b^Significant differences within the same row (*p* < 0.05).

a CON, control group (*n* = 8, no zinc proteinate or zinc oxide supplementation); L-ZnP, low-zinc proteinate group (*n* = 8, 261 mg/day of zinc proteinate); M-ZnP, medium-zinc proteinate group (*n* = 8, 523 mg/day of zinc proteinate); H-ZnP, high-zinc proteinate group (*n* = 8, 784 mg/day of zinc proteinate); ZnO, zinc oxide group, (*n* = 8, 232 mg/day of zinc oxide).

### 3.5. Rectal microbiota composition at the genus level

The effects of different zinc sources on the rectal microbial composition at the genus level on day 70 are shown in Table 7. *Faecalibacterium*, *Bacteroidaceae_Bacteroides*, *Prevotella*, and *Blautia* were the dominant genera, followed by *Lactobacillus*, *Oscillospira*, *Subdoligranulum*, *Odoribacte*, and *Dorea* (Table 7). Early supplementation with ZnO increased the relative abundances of *Prevotella* (*p* < 0.05), *Subdoligranulum* (*p* < 0.05), and *Odoribacter* (*p* < 0.05) compared with the CON group. There were no differences in the composition of rectal microflora at the genus level among the three ZnP groups and the CON group, except that the relative abundance of Odoribacter was higher in the H-ZnP group than in the CON group (*p* < 0.05).

**Table 7:** Analysis of rectal microflora composition at the genus level in preweaned Holstein dairy calves on day 70.

Genus, %	Treatment^a^					SEM	*p*-value
	CON	L-ZnP	M-ZnP	H-ZnP	ZnO		
*Faecalibacterium*	11.01	13.76	16.50	17.27	9.85	0.10	0.535
*Bacteroidaceae_Bacteroides*	12.29	27.38	20.01	15.35	26.26	0.13	0.077
*Prevotella*	11.92^b^	9.36^b^	12.66^ab^	7.51^b^	25.46^a^	0.14	0.032
*Blautia*	9.14	2.92	3.06	4.83	3.96	0.06	0.533
*Lactobacillus*	0.37	3.38	2.57	0.50	1.15	0.04	0.795
*Oscillospira*	1.39	3.08	3.18	2.34	2.34	0.02	0.072
*Subdoligranulum*	0.38^b^	0.58^ab^	0.47^b^	0.75^ab^	2.32^a^	0.01	0.028
*Odoribacter*	0.21^b^	0.16^b^	0.61^ab^	0.73^a^	0.67^a^	0.01	0.044
*Dorea*	1.42	0.93	0.72	1.00	0.70	0.01	0.842

^a,b^Significant differences within the same row (*p* < 0.05).

a CON, control group (*n* = 8, no zinc proteinate or zinc oxide supplementation); L-ZnP, low-zinc proteinate group (*n* = 8, 261 mg/day of zinc proteinate); M-ZnP, medium-zinc proteinate group (*n* = 8, 523 mg/day of zinc proteinate); H-ZnP, high-zinc proteinate group (*n* = 8, 784 mg/day of zinc proteinate); ZnO, zinc oxide group, (*n* = 8, 232 mg/day of zinc oxide).

### 3.6. Correlation of rectal microbiota with growth performance, antioxidant indicators, and plasma zinc concentrations

**Figure 3:**
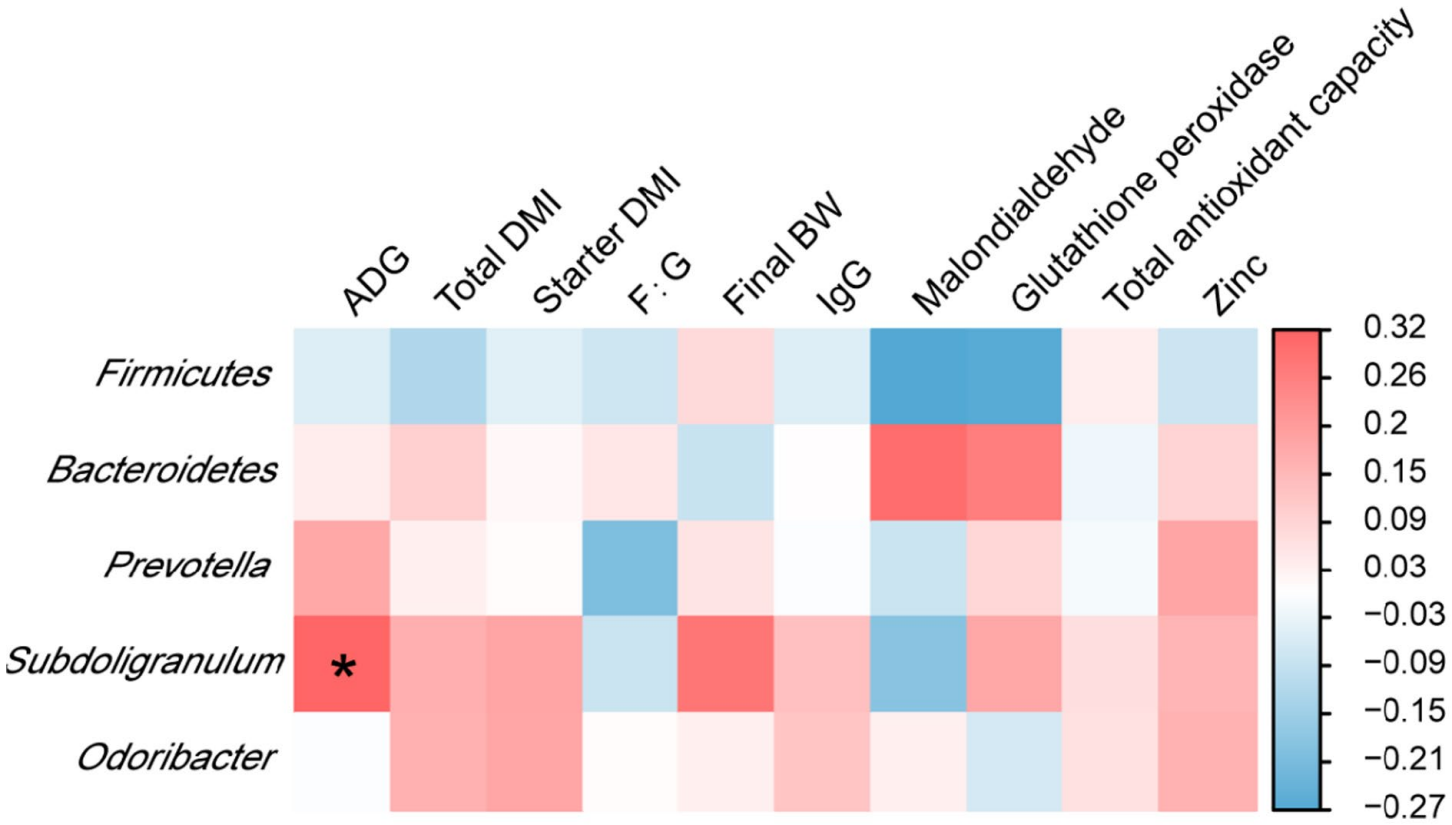
Interaction heatmaps showing the associations between the relative abundances of rectal bacterial phyla and genera and the growth performance, antioxidant indicators, and plasma zinc concentrations of Holstein dairy calves on day 70. ADG, average daily gain; total DMI, total dry matter intake; starter DMI, starter dry matter intake; F:G, feed to gain; final BW, final body weight; IgG, immunoglobulin G.

Analyses of the correlation of rectal microbiota with the growth performance, antioxidant indicators, and plasma zinc concentrations of calves on day 70 showed that the relative abundance of *Subdoligranulum* was positively associated with ADG (*p* < 0.05) (Figure 3).

## 4. Discussion

Zinc is an essential trace mineral for humans and animals with effective antidiarrheal and growth promotion activities (28, 29). Lower doses of zinc, especially in organic forms, can reduce diarrhea and promote the growth of young animals (10, 15, 25). In agreement with these findings, the present study found that dietary supplementation with ZnP linearly increased total DMI, ADG, and feed efficiency, thus increasing the final BW of preweaned dairy calves. Furthermore, compared with the control group, supplementation with ZnP to yield 80 mg/day of zinc significantly enhanced the growth performance of dairy calves (15). In the present study, supplementation with ZnP to yield 80 mg/day zinc exhibited growth promotion effects similar to that of ZnO yielding 180 mg/day zinc, except for the final BW. Besides, the incidence of diarrhea is also a factor in evaluating the health status of calves. Diarrhea occurs frequently during the first month of life (30). The present study found that the incidence of diarrhea declined as the calves grew older. The incidence of diarrhea in the CON group fluctuated from 4.17 to 18.2 during the experimental period and peaked during the first 28 days of life, during which supplementation with ZnP and ZnO significantly reduced the incidence of diarrhea. Notably, calves in the M-ZnP and ZnO groups showed significant reductions in the incidence of diarrhea compared with the CON group on days 1–28, and 29–56. Overall, considering the source and concentrations of ZnP, the study suggests that dietary zinc supplementation to preweaned dairy calves in the form of ZnP (80 mg/day zinc), can positively boost their growth performance by reducing the occurrence of diarrhea.

Zinc is essential for the function of the immune system, thus affecting cells of both the innate and adaptive immune systems (31, 32). Zinc deficiency has been shown to impair immune function, which can be restored and even improved by zinc supplementation, especially during early life (25, 33, 34). Supplementation with ZnO was found to increase serum concentrations of IgG in piglets 14 days after weaning but did not affect serum antibody concentrations 28 days after weaning (35). Similarly, in the present study, dietary supplementation with ZnP or ZnO did not affect immunoglobulin indices of preweaned dairy calves. This may be due to the relatively long experimental period of the present study. However, the antidiarrheal effect of dietary zinc has been associated with its immune effect (25, 28, 36), and dietary zinc supplementation has been reported to improve immune function by increasing the serum IgG content of calves (37). The findings of the present study, showing that serum IgG concentrations increased linearly with ZnP dose, were consistent with these earlier results.

Reactive oxygen species, which are generated during normal cellular respiration, can be scavenged by the free radical scavenging system (38). Zinc acts as an antioxidant by promoting the free radical scavenging system in the body (39). The SOD is involved in the cellular scavenging of free radicals and reactive oxygen species (40). In this study, serum SOD activities were higher in the M-ZnP and ZnO groups than in the CON group. This aligned with the results showing that dietary supplementation with 30 mg/kg zinc DM in the form of ZnP or zinc sulfate (ZnSO_4_) enhanced serum SOD activities in lambs (41). Furthermore, the harmful effects of reactive oxygen species accumulation can be prevented by GSH-Px (39), which metabolizes hydrogen peroxide and lipid peroxides to water and harmless oxy compounds (42). The present study found that serum GSH-Px activities were increased by dietary supplementation with ZnP or ZnO, with increasing levels of ZnP supplementation associated with a linear increase in serum GSH-Px activity. T-AOC is an antioxidant biomarker (43), whereas MDA is an end-product of lipid peroxidation regarded as a marker of oxidative stress and antioxidant status (44). In the present study, supplementation with ZnP linearly increased serum T-AOC activity and decreased serum MDA concentration, indicating that ZnP enhanced antioxidant activity in dairy calves.

Plasma trace element concentration is the most direct reflection of body trace element status, with alterations in plasma zinc concentration being highly sensitive to changes in dietary zinc content (45). Dietary supplementation with Zn-Met or ZnO during the first 2 weeks of life increased plasma zinc concentrations in calves (26). The present study found that increases in plasma zinc concentrations in dairy calves correlated with increasing doses of ZnP supplement, and that supplementation with ZnP to yield ≥80 mg/day zinc was associated with significantly higher plasma zinc concentrations compared with the CON group. Although different sources of zinc did not affect plasma zinc concentrations, the dose of ZnO was relatively higher than that of ZnP, suggesting that the bioavailability of ZnP was higher than that of ZnO. This finding was in good agreement with previous studies showing that organic zinc had higher bioavailability than inorganic zinc (26, 46).

Zinc has been shown to alter the structure of gut microbes (47–49). In the present study, the dominant phyla in the rectum of dairy calves from birth to day 70 were Firmicutes and Bacteroidetes, consistent with previous findings (50, 51). Furthermore, the relative abundance of Firmicutes in the ZnO group was lower on day 70, which may be due to the bacteriostatic effects of zinc ions in the intestinal lumen (48, 52). Zinc proteinate was absorbed in its intact form and therefore did not affect Firmicutes (53). Interestingly, some bacteria have strong tolerance to zinc ions (e.g., *Bacteroides* and *Prevotella*, belong to Bacteroidetes) (49, 52), which may explain the greater relative abundance of Bacteroidetes in the ZnO group on day 70. Bacteroidetes generally produce butyrate, which has antitumor properties, presents the main energy source for enterocytes, and enhances intestinal health by maintaining mucosal integrity (54, 55). In the present study, supplementation with ZnO increased the abundance of Bacteroidetes, improving intestinal health. *Faecalibacterium*, *Bacteroidance-Bacteroides*, and *Prevotella* were the main intestinal genera from day 1 to day 70, a finding consistent with previous results (50, 56). *Prevotella* protects mucosal and epithelial tissues by inducing lymphocytes to secrete cytokines (57). *Subdoligranulum* is contributing to increasing ADG, with the relative abundance of *Subdoligranulum* positively correlating with ADG (58). Consistent with these reports, the present study found that the relative abundances of *Prevotella* and *Subdoligranulum* were higher in the ZnO group than in the CON group, which may have contributed to the higher ADG in ZnO-treated dairy calves. ZnP supplementation increased the relative abundance of *Odoribacter*, which may help improve the intestinal health of dairy calves, as *Odoribacter* is a type of beneficial bacterium that can also produce butyrate (59).

## 5. Conclusion

The present study showed that early supplementation with ZnP or ZnO to yield more than 80 mg/day zinc during the first 2 weeks of life reduced neonatal diarrhea from day 1 to day 28, and increased the DMI, ADG, and feed efficiency of preweaned dairy calves. Furthermore, supplementation with ZnP linearly increased serum IgG concentration, GSH-Px and T-AOC activities, and plasma zinc content but reduced serum MDA concentration. The effects of supplementation with ZnP to yield 80 mg/day zinc were similar to the effects of supplementation with ZnO to yield 180 mg/day zinc, except that the final BW of calves was higher in the ZnO group. Early supplementation with ZnP did not affect rectal microbial diversity or composition, whereas ZnO reduced the relative abundance of the phylum Firmicutes while increasing the relative abundance of the phylum Bacteroidetes, and enhanced the abundance of the genera *Prevotella*, *Subdoligranulum*, and *Odoribacter*. This study demonstrated that ZnP is a potential organic source of zinc with greater bioavailability than ZnO for preweaned dairy calves. Zinc proteinate yielding doses of 80 mg/day zinc is recommended for early dietary supplementation.

## Data availability statement

The datasets presented in this study can be found in online repositories. The names of the repository/repositories and accession number(s) can be found in the article/supplementary material.

## Ethics statement

The animal study was approved by the Chinese Academy of Agricultural Sciences Animal Care and Use Committee. The study was conducted in accordance with the local legislation and institutional requirements.

## Author contributions

JL and PS designed the experiment. JL, XY, FM, YW, and YJ carried out the experiment. JL and XY wrote the manuscript. NH and PS revised the manuscript. All authors contributed to the article and approved the submitted version.

## Funding

This study was financially supported by the National Key Research and Development Program of China (2022YFD1300505 and 2022YFD1301101), the earmarked fund for China Agriculture Research System (CARS-37), and the Agricultural Science and Technology Innovation Program (cxgc-ias-07, Beijing, China).

## Conflict of interest

The authors declare that the research was conducted in the absence of any commercial or financial relationships that could be construed as a potential conflict of interest.

## Publisher’s note

All claims expressed in this article are solely those of the authors and do not necessarily represent those of their affiliated organizations, or those of the publisher, the editors and the reviewers. Any product that may be evaluated in this article, or claim that may be made by its manufacturer, is not guaranteed or endorsed by the publisher.

## Footnotes

^1^ https://www.Genescloud.cn
